# Supplementary figures and images for: Mammalian cells internalize bacteriophages and use them as a resource to enhance cellular growth and survival
Source: PLoS Biol. 2023 Oct 26;21(10):e3002341. doi: 10.1371/journal.pbio.3002341 (PMC10602308; doi:10.1371/journal.pbio.3002341)

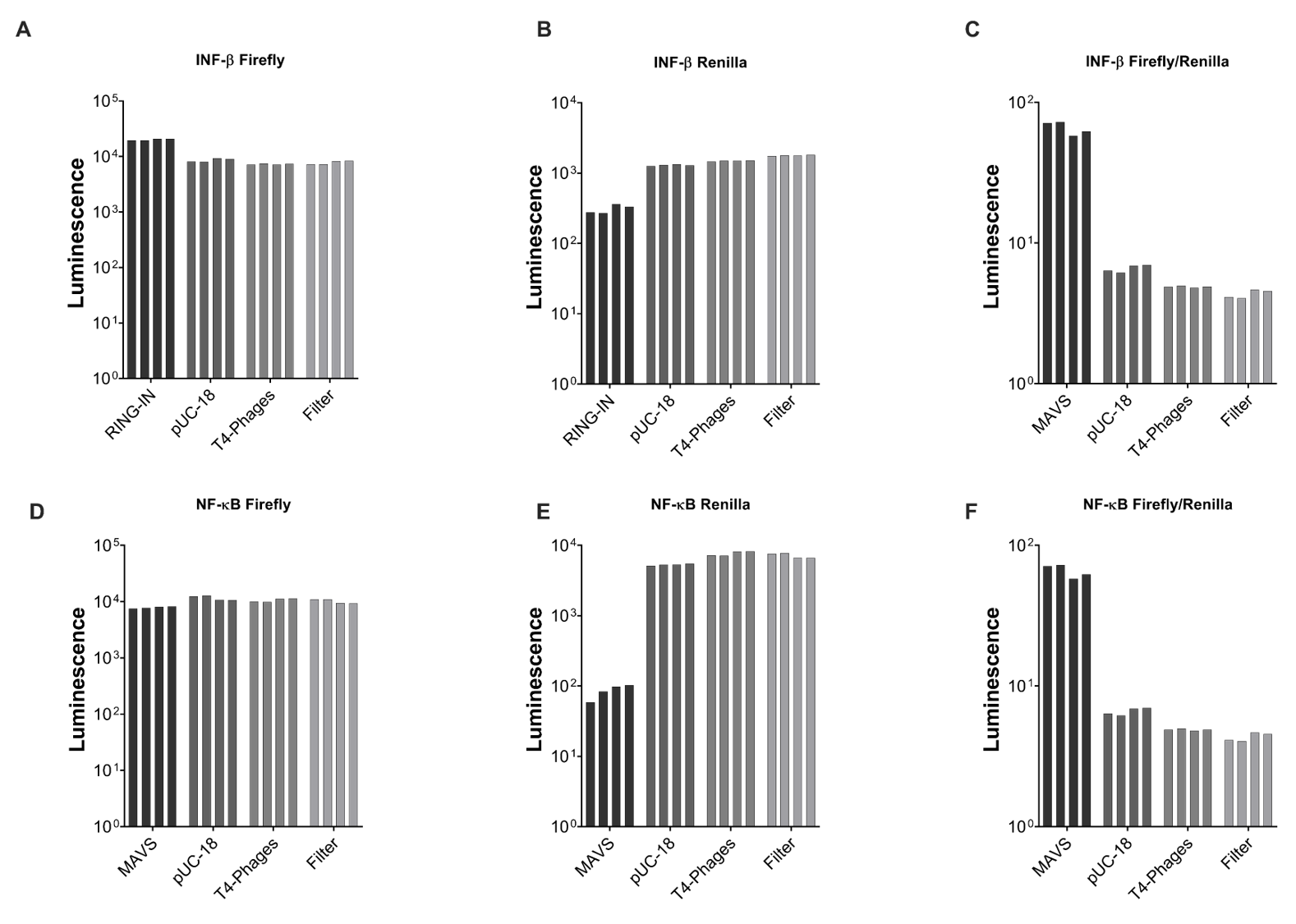

Supplement: S1 Fig — Individual results from the luciferase assay with IFN-ß and NF-κB from the Firefly and Renilla reporter. (A) Firefly IFN-ß luminescence values from each well. (B) Renilla IFN-ß luminescence values from each well. (C) IFN-ß normalized luminescence values, Firefly/Renilla, from each well. (D) Firefly NF-κB luminescence values from each well. (E) Renilla NF-κB luminescence values from each well. (F) NF-κB normalized luminescence values, Firefly/Renilla, from each well. Raw data can be found in S3 Data. (TIF) [file pbio.3002341.s001.tif]

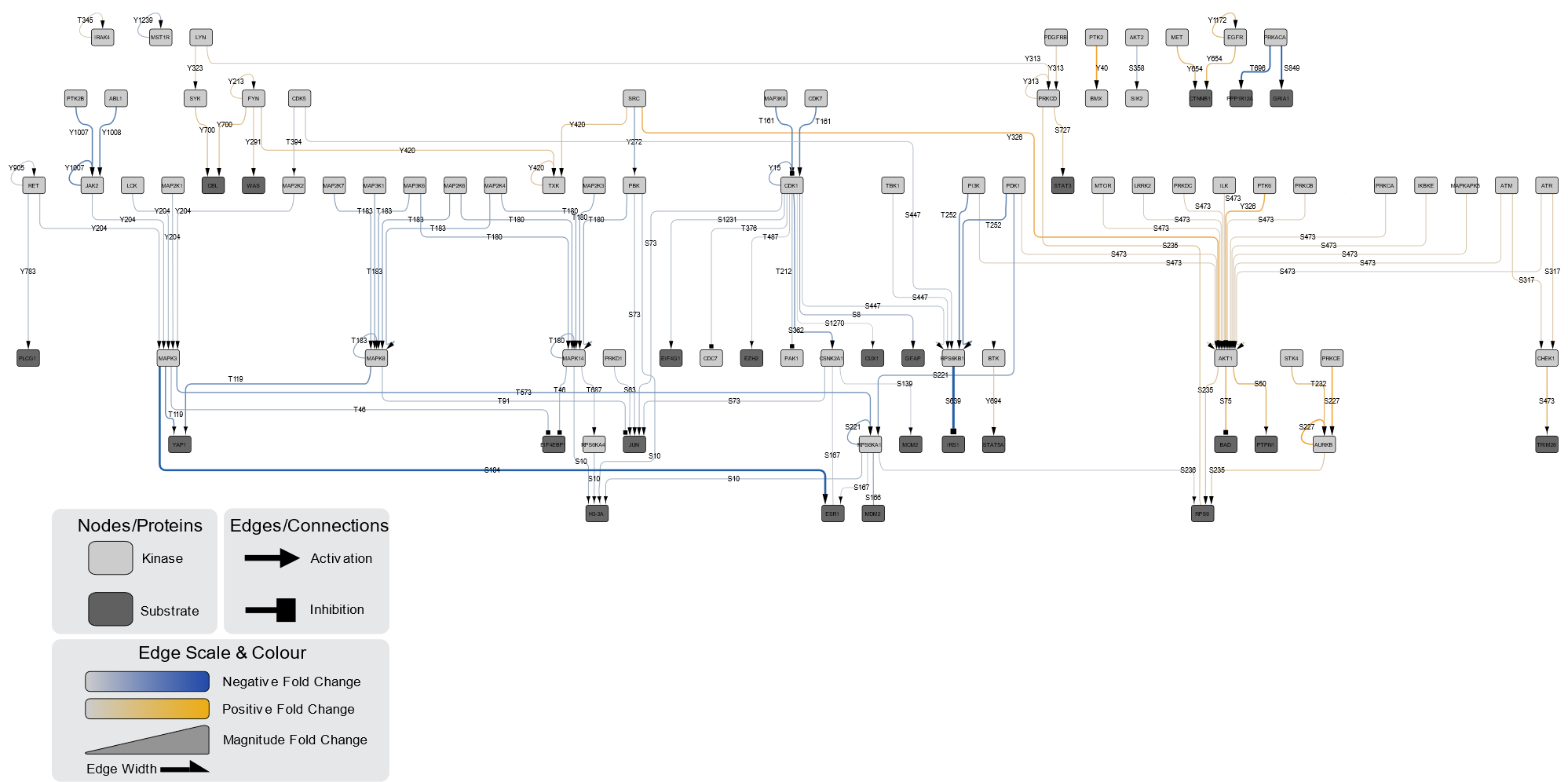

Supplement: S2 Fig — Showing all the interactions detected in the microarray. (TIF) [file pbio.3002341.s002.tif]

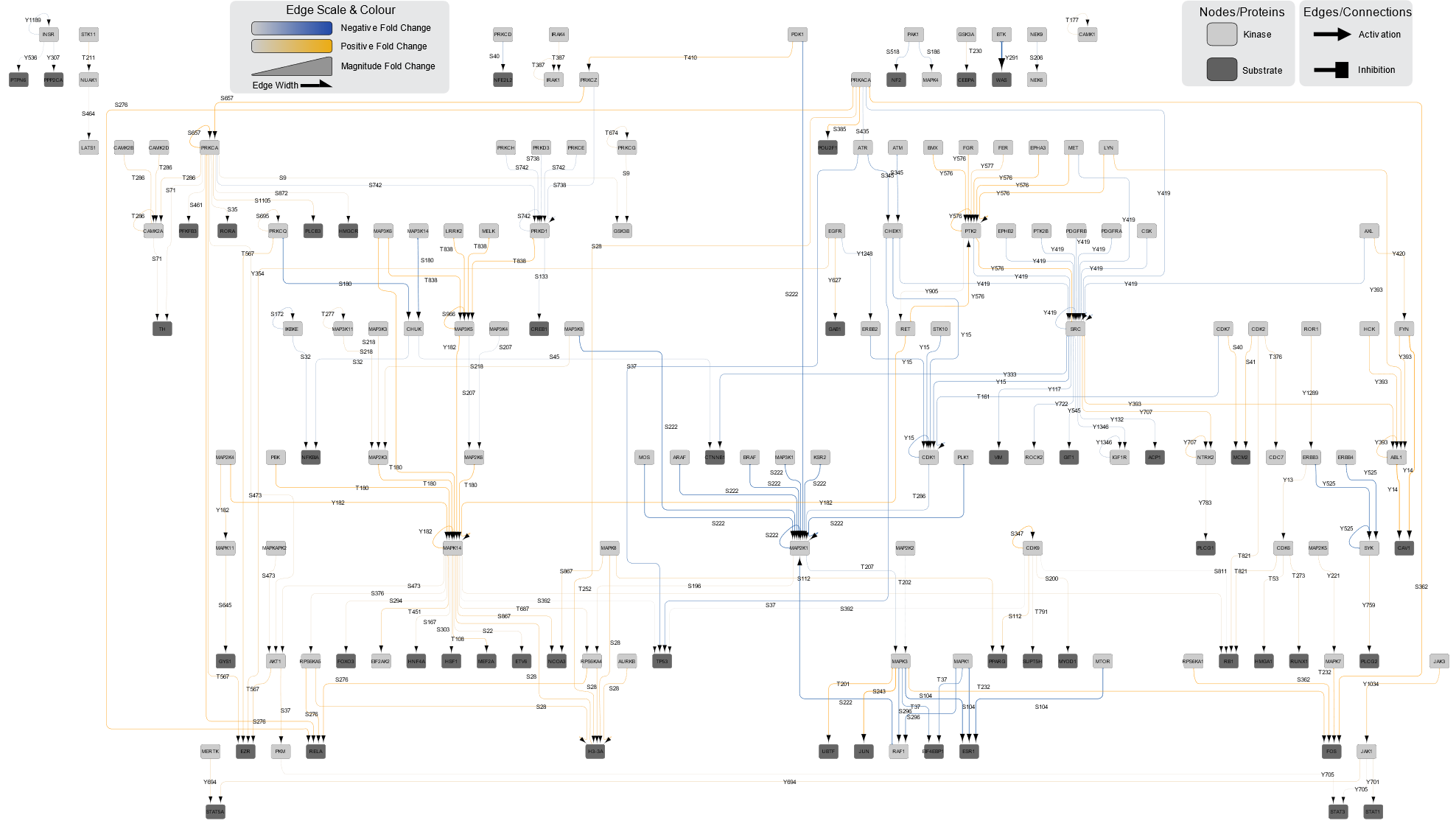

Supplement: S3 Fig — Showing all the interactions detected in the microarray. (TIF) [file pbio.3002341.s003.tif]

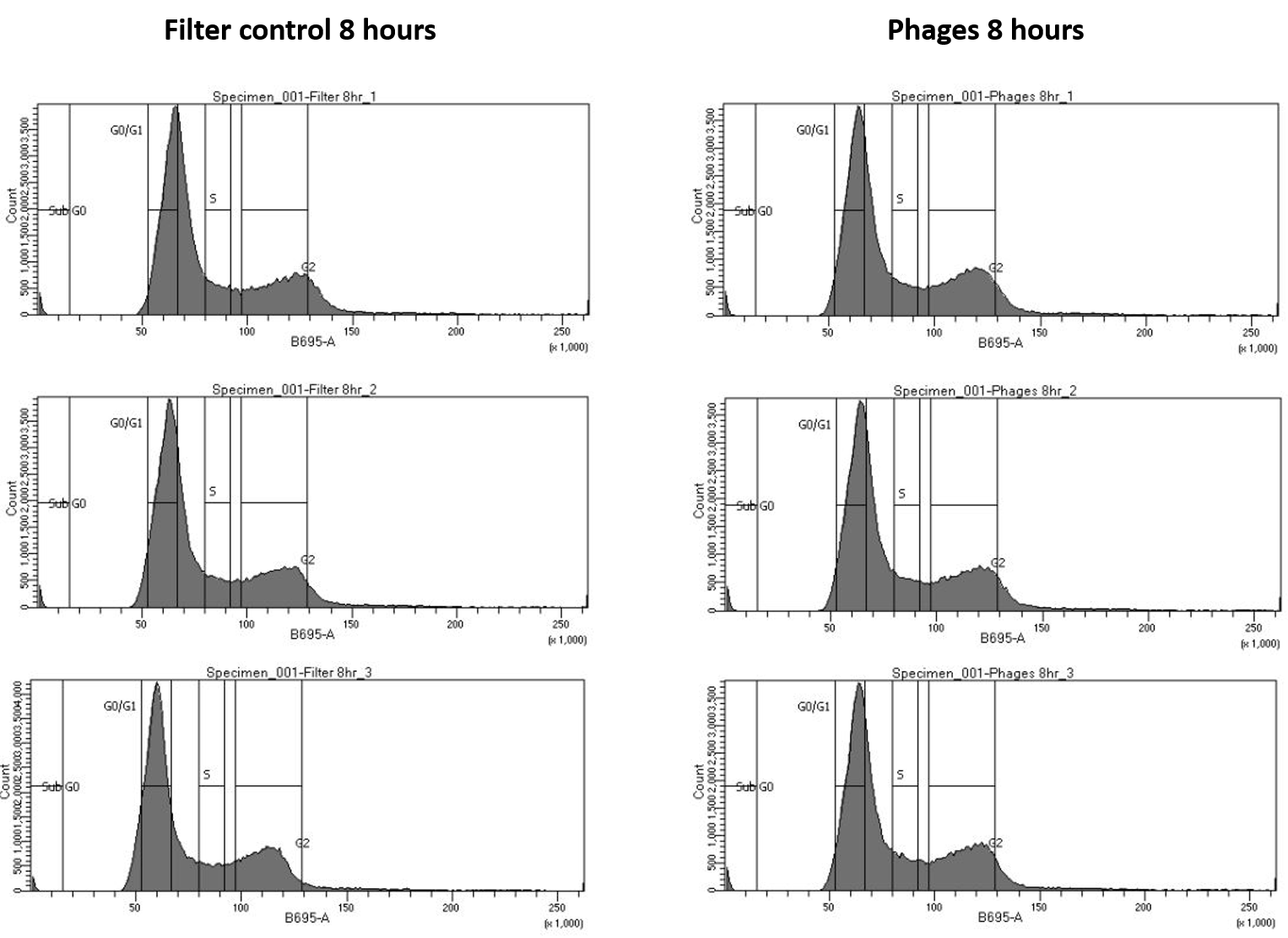

Supplement: S4 Fig — On the left, the 3 control samples were incubated with the Filter control for 8 hours. On the right, the 3 samples were incubated with the T4 phages for 8 hours. The cell cycle stages were set on a nonincubated sample and kept fixed for all the following analyses. (TIF) [file pbio.3002341.s004.tif]

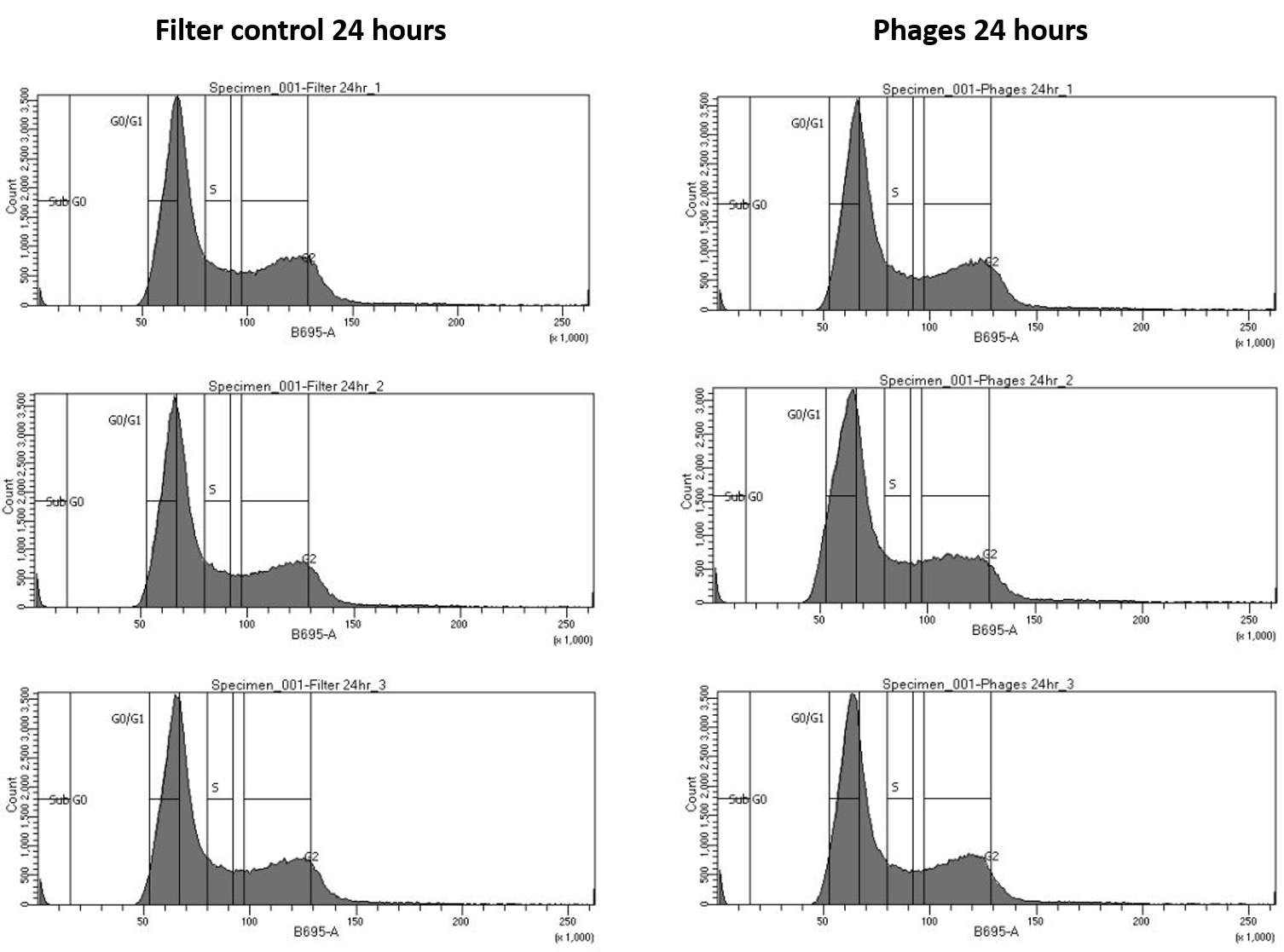

Supplement: S5 Fig — On the left, the 3 control samples were incubated with the Filter control for 24 hours. On the right, the 3 samples were incubated with the T4 phages for 24 hours. The cell cycle stages were set on a nonincubated sample and kept fixed for all the following analyses. (TIF) [file pbio.3002341.s005.tif]
