# Supplementary material for: Mammalian cells internalize bacteriophages and use them as a resource to enhance cellular growth and survival
Source: PLoS Biol. 2023 Oct 26;21(10):e3002341. doi: 10.1371/journal.pbio.3002341 (PMC10602308; doi:10.1371/journal.pbio.3002341)
Supplement: S1 Table — (DOCX) [file pbio.3002341.s009.docx]

**Table S1: Table listing the mains leads for the microarray MDCK-I sample.**

| **Antibody ID No.** | **Target Name** | **Antibody P-Site** | **% CFC** |
| --- | --- | --- | --- |
| NN430-1 | Myc | Pan-specific | 371 |
| PN274 | STAT4 | S721 | 179 |
| PN538 | STAM2 | Y374 | 139 |
| PK833 | TRIM28 (TIF1B) | S473 | 116 |
| PK531 | AurKB (Aurora B, AIM-1) | T232 | 93 |
| NK284-1 | PBK | Pan-specific | 87 |
| PN671 | STAT5A | Y694 | 81 |
| PK607 | EphA2 | Y772 | 80 |
| NK273-1 | IRR (INSRR) | Pan-specific | 66 |
| PK817 | SMG1 | T3550 | 54 |
| NK120-7 | p38a MAPK (MAPK14) | Pan-specific | 53 |
| NK121-3 | p38d MAPK (MAPK13) | Pan-specific | 45 |
| PN501 | ACTB | Y53 | 44 |
| NK121-2 | p38d MAPK (MAPK13) | Pan-specific | 40 |
| PK670 | JNK1 (MAPK8) | Y185 | 39 |
| PN655 | SIN3A | S832 | 36 |
| NK181-3 | TYK2 | Pan-specific | 32 |
| PK536 | GRK2 (BARK1, ADRBK1) | S670 | 32 |
| PK836 | TRIM33 (TIF1G) | S1119 | 31 |
| NK250-2 | SIK3 (QSK) | Pan-specific | 28 |
| PK665 | IRAK4 | T345+S346 | 26 |
| NK076-5 | IKKb (IkBKB) | Pan-specific | 23 |
| PN638 | p53 (TP53) | T18+S20 | 20 |
| PK745 | p70S6K (S6Ka, RPS6KB1) | T412 | 14 |
| PK786 | PRP4K (PRP4, PRPF4B) | Y849 | 11 |
| PK645 | Fyn | Y531 | -5 |
| NK255-3 | WNK4 (PRKWNK4) | Pan-specific | -8 |
| PP505 | PPP2CB | T304 | -10 |
| NK156-6 | Raf-B (BRaf) | Pan-specific | -10 |
| NK269-2 | Frk | Pan-specific | -11 |
| PN644 | PPARg-1 | S112 | -12 |
| PN583 | ERa (ESR1) | S167 | -13 |
| NK107-4 | MEKK1 (MAP3K1) | Pan-specific | -13 |
| NK026-6 | CDK2 | Pan-specific | -15 |
| PN745 | CTNNB1 | Y489 | -15 |
| PK879 | ERK1 (MAPK3) | S283 | -15 |
| PK608 | EphA3 | Y779 | -16 |
| PK666 | ITK | Y512 | -16 |
| PN110 | CRYAB | S45 | -17 |
| PN574 | BCLAF1 | S512 | -17 |
| NK030-2 | CDK7 | Pan-specific | -19 |
| PN512 | ENO2 | Y25 | -20 |
| PK558 | CDC7 | T376 | -22 |
| PK793 | Ret (GDNF receptor) | Y905 | -23 |
| PN002 | ACC1 (ACACA) | S80 | -23 |
| NK052-5 | EGFR (ErbB1) | Pan-specific | -26 |
| PN590 | FOXK1 | S441+S445 | -31 |
| PK570 | CDK5 | Y15 | -34 |
| NP001 | CD45 (PTPRC; Receptor-type tyrosine-protein phosphatase C) | Pan-specific | -38 |
| PN196 | GATA1 | S142 | -40 |
| PN144 | PLCG1 | Y783 | -42 |
