# Supplementary material for: Mammalian cells internalize bacteriophages and use them as a resource to enhance cellular growth and survival
Source: PLoS Biol. 2023 Oct 26;21(10):e3002341. doi: 10.1371/journal.pbio.3002341 (PMC10602308; doi:10.1371/journal.pbio.3002341)
Supplement: S2 Table — (DOCX) [file pbio.3002341.s010.docx]

**Table S2: Table listing the mains leads for the microarray A549 sample.**

| **Antibody ID No.** | **Target Name** | **Antibody P-Site** | **Log2 Fold Change** |
| --- | --- | --- | --- |
|  |  |  |  |
| sc-7263 | Wip1 (PPM1D) | Pan-specific | 2,26 |
| NK116-3 | mTOR (FRAP) | Pan-specific | 2,12 |
| NK225-2 | MEKK6 (MAP3K6; ASK2) | Pan-specific | 1,68 |
| PN827 | Huntingtin (HTT) | Pan+S13+S16 | 1,57 |
| sc-6241 | HSP105 (HSPH1; HSP110) | Pan-specific | 1,52 |
| NK165-3 | RSK2 (RPS6KA3) | Pan-specific | 1,52 |
| sc-639 | PKD1 (PRKCM; PKCm; PRKD1) | Pan-specific | 1,51 |
| PK917 | TrkB (NTRK2) | Y706+Y707 | 1,49 |
| KAP-ST205 | IRAK2 | Pan-specific | 1,42 |
| sc-7973 | p38a MAPK (MAPK14; CSBP; MXI2; SAPK2a) | Y182 | 1,41 |
| NNCOV2S-5 | SARS-CoV-2 Spike RBD | Pan-specific | 1,41 |
| 07-191 | H2B (Histone H2B) | S15 | 1,39 |
| NK229-2 | MELK | Pan-specific | 1,39 |
| PK882 | GSK3a | Y279 | 1,37 |
| PK910 | PKR1 (PRKR; EIF2AK2) | T451 | 1,34 |
| sc-639 | PKD1 (PRKCM; PKCm; PRKD1) | Pan-specific | 1,29 |
| PK893 | GCK (Glucokinase) | Pan+S411 | 1,29 |
| PK537 | GRK2 (BARK1; ADRBK1) | Y356 | 1,28 |
| NK004-3 | CDK15 (PFTAIRE2; ALS2CR7) | Pan-specific | 1,28 |
| PN629 | NCOA3 (SRC-3) | S867 | 1,26 |
| PK571 | CDK6 | Y13 | 1,25 |
| NK105-4 | MKK6 (MAP2K6; MEK6) | Pan-specific | 1,25 |
| NK104-5 | MEK5 (MAP2K5; MKK5) | Pan-specific | 1,24 |
| PN700 | FBPase 2 (FBP2) | Y216 | 1,23 |
| PN824 | H2AFX (H2AX; Histone H2A.X) | S140 | 1,22 |
| PN644 | PPARg-1 | S112 | 1,21 |
| NN265-1 | Grp170 (HYOU1; ORP-150) | Pan-specific | 1,21 |
| PK647 | GSK3a | S278+Y279 | 1,21 |
| NNCOV2S-9 | SARS-CoV-2 Spike S2 | Pan-specific | 1,20 |
| PN782 | UGDH | Y352 | 1,18 |
| PK712 | Met (HGF receptor) | Y1234+Y1235 +S1236 | 1,16 |
| NK116-5 | mTOR (FRAP) | Pan-specific | 1,16 |
| 44-956 | PKCg (PRKCG) | T514 | 1,15 |
| NK225-3 | MEKK6 (MAP3K6; ASK2) | Pan-specific | 1,12 |
| 05-184 | Src | Pan-specific | 1,12 |
| PK567 | CDK12 (Cdc2L7; CRK7) | T893 | 1,11 |
| NK107-3 | MEKK1 (MAP3K1) | Pan-specific | 1,10 |
| PN772 | PKM2 | Y105 | 1,10 |
| PK574 | CDK9 | S347 | 1,07 |
| PN718 | PKM2 | S37 | 1,07 |
| PN703 | GYS1 | S641+S645 | 1,07 |
| NK025-7 | CDK1 (CDC2) | Pan-specific | 1,06 |
| PK729 | mTOR (FRAP) | S2448 | 1,06 |
| NK237-2 | ATR | Pan-specific | 1,05 |
| NK059-5 | p38g MAPK (MAPK12; ERK6; SAPK3) | Pan-specific | 1,04 |
| PK606 | EphA2 | Y588 | 1,03 |
| NN060-12 | Hsc70 (HSPA8; Hsc70; HSP73; HSPA10) | Pan-specific | 1,03 |
| NK112 | Mos | Pan-specific | 1,03 |
| NK280-1 | MLK4 (MAP3K21) | Pan-specific | 1,03 |
| sc-6212 | Ksr1 | Pan-specific | 1,02 |
| PN719 | PLCB3 | S1105 | 1,02 |
| NK283-1 | MRCKb (CDC42BPB) | Pan-specific | 1,02 |
| AP7642a | VEGFR1 (Flt1) | Pan-specific | 1,01 |
| NK033-3 | CDK10 (PISSLRE) | Pan-specific | 1,01 |
| NK269-2 | Frk | Pan-specific | 1,01 |
| PK770 | PKD1 (PRKCM; PKCm; PRKD1) | S205 | 1,01 |
| 06-0032 | STAT5A | Y694 | 1,00 |
| NK275-1 | MARK2 | Pan-specific | -1,00 |
| 9111 | CDK1 (CDC2) | Y15 | -1,00 |
| PN637 | TP53 (p53) | S6+S9 | -1,01 |
| PN630 | NFAT1 | S217+S221 | -1,02 |
| PN710 | NMDAR2A NMDA (GRIN2A; Glutamate [NMDA] receptor subunit epsilon-1) | Y943 | -1,02 |
| PN514 | ESYT1 | Y822 | -1,02 |
| PN584 | ERF | T526 | -1,03 |
| PN513 | ERBB2IP (Erbin) | Y1104 | -1,03 |
| NNCOV2S-16 | SARS-CoV-2 Spike S2 | Pan-specific | -1,05 |
| AP7611b | EphA6 | Pan-specific | -1,05 |
| PK624 | ERK4 (MAPK4) | S186 | -1,06 |
| NP008-2 | DUSP2 (PAC1) | Pan-specific | -1,06 |
| PN864 | ARID1A | Y1508 | -1,07 |
| PK792 | Raf1 (c-Raf; RafC) | S301+T303 | -1,07 |
| PN698 | FASN (FAS) | S207 | -1,08 |
| PK638 | Fgr | Y208+Y209 | -1,09 |
| PK747 | p70S6Kb (S6Kb2; RPS6KB2) | S423 | -1,10 |
| PK880 | ERK2 (MAPK1; ERT1) | Y263+S266 | -1,11 |
| NK120-8 | p38a MAPK (MAPK14; CSBP; MXI2; SAPK2a) | Pan-specific | -1,11 |
| sc-7439 | Nek2 | Pan-specific | -1,12 |
| 11097 | TP53 (p53) | S33 | -1,12 |
| PK736 | NLK | T298 | -1,14 |
| 07-012 | FAK (PTK2) | Y397 | -1,15 |
| AP7518b | CDK2 | Pan-specific | -1,16 |
| PK866 | ERK1 (MAPK3; ERT2) | Y204+T207 | -1,16 |
| PN761 | NF1 | Y2577 | -1,16 |
| NK019-3 | CAMK2d | Pan-specific | -1,17 |
| NK059-4 | p38g MAPK (MAPK12; ERK6; SAPK3) | Pan-specific | -1,18 |
| AAP-104 | CASP4 (Caspase 4) | Pan-specific | -1,18 |
| PN709 | NF2 | S518 | -1,19 |
| NK085-4 | JAK2 | Pan-specific | -1,19 |
| NN604-2 | NRP1 | Pan-specific | -1,21 |
| AP7802b | IRAK1 | Pan-specific | -1,22 |
| NK231 | ErbB3 (HER3) | Pan-specific | -1,22 |
| NN454-1 | 14-3-3-S (YWHAS; SFN) | Pan-specific | -1,22 |
| PN631 | NFAT3 (NFATc4) | S213+S217 | -1,23 |
| DB033 | NFKB p65 (Rel A) | Pan-specific | -1,23 |
| PN841 | NMDAR1 (NR1) | S897 | -1,28 |
| PK743 | p38d MAPK (MAPK13) | Y182 | -1,28 |
| NN300-1 | NrCAM | Pan-specific | -1,28 |
| 11134 | CDK1 (CDC2) | T161 | -1,28 |
| AP7612a | EphA7 | Pan-specific | -1,30 |
| PK873 | Abl (Abl1) | Y393+T394 | -1,30 |
| 11063 | EZR (Ezrin; VIL2) | Y354 | -1,30 |
| PK629 | FAK (PTK2) | Y577 | -1,31 |
| NK120-10 | p38a MAPK (MAPK14; CSBP; MXI2; SAPK2a) | Pan-specific | -1,33 |
| C27220 610266 | CD45 (PTPRC; Receptor-type tyrosine-protein phosphatase C) | Pan-specific | -1,33 |
| 11098 | TP53 (p53) | S37 | -1,35 |
| sc-7230 | ATM | Pan-specific | -1,35 |
| PK879 | ERK1 (MAPK3; ERT2) | S283 | -1,36 |
| sc-1214 | ATM | Pan-specific | -1,37 |
| NN296-1 | NLGN4x (NLGN4l) | Pan-specific | -1,37 |
| NK121-4 | p38d MAPK (MAPK13) | Pan-specific | -1,39 |
| PN842 | NRF2 | S40 | -1,42 |
| AP7500a | ERK1 (MAPK3; ERT2) | Pan-specific | -1,44 |
| PK627 | FAK (PTK2) | Y397 | -1,45 |
| PK886 | MEK1 (MAP2K1; MKK1) | T286 | -1,46 |
| NN229-1 | CD74 | Pan-specific | -1,50 |
| PP527 | CD45 (PTPRC; Receptor-type tyrosine-protein phosphatase C) | Y1216 | -1,50 |
| NN451-3 | 14-3-3e (YWHAE) | Pan-specific | -1,52 |
| AP7504a | ERK5 (MAPK7; BMK) | Pan-specific | -1,54 |
| PK865 | ERK1 (MAPK3; ERT2) | T207 | -1,56 |
| NN230-1 | CDC37 | Pan-specific | -1,61 |
| PK878 | ERK1 (MAPK3; ERT2) | S265 | -1,62 |
| PK558 | CDC7 | T376 | -1,65 |
| NP038-1 | CDC25A | Pan-specific | -1,66 |
| 44-864 | Integrin a4 - pS1021 | S1021 | -1,68 |
| NP038-3 | CDC25A | Pan-specific | -1,76 |
| PK888 | p38d MAPK (MAPK13) | S261+T265 | -1,80 |
| DB040 | Trail | Pan-specific | -1,81 |
| C25820 610250 | CDC34 | Pan-specific | -1,88 |
| IMG-139 | TBK1 (IKKd) | Pan-specific | -1,88 |
| NN204-1 | ATAD1 ATPase | Pan-specific | -1,94 |
| PK791 | Raf1 (c-Raf; RafC) | S296 | -2,02 |
| PN521 | ITSN2 | Y968 | -2,03 |
| PK668 | JAK2 | Y570 | -2,07 |
| NK059-3 | p38g MAPK (MAPK12; ERK6; SAPK3) | Pan-specific | -2,15 |
| NK020-1 | CaMK2g | Pan-specific | -2,18 |
| sc-1284 | ERK5 (MAPK7; BMK) | Pan-specific | -2,19 |
| NN205-2 | Ataxin 1 (Atxn1; SCA1) | Pan-specific | -2,19 |
| PK555 | CaMK2a (CaMKII) | T286 | -2,28 |
| NP038-2 | CDC25A | Pan-specific | -2,29 |
| 13-9800 | CAMK2b | Pan-specific | -2,32 |
| DB075 | IkBa (MAD3; IkBa) | Pan-specific | -2,49 |
| sc-58758 | EZR (Ezrin; VIL2) | Pan-specific | -2,52 |
| sc-1285 | ERK5 (MAPK7; BMK) | Pan-specific | -2,54 |
| NK055-2 | ERK1 (MAPK3; ERT2) | Pan-specific | -2,60 |
| sc-56070 | CASP8 (Caspase 8) | Pan-specific | -2,75 |
| 13-9800 | CAMK2b | Pan-specific | -2,95 |
| NK084-3 | JAK1 | Pan-specific | -3,00 |
| sc-56063 | CASP7 p20 (Caspase 7) | Pan-specific | -3,03 |
| PK659 | IKKa (IkBKA) | T179+S180 | -3,55 |
| sc-622 | CASP1 (Caspase-1) | Pan-specific | -4,17 |
