# Supplementary material for: Mammalian cells internalize bacteriophages and use them as a resource to enhance cellular growth and survival
Source: PLoS Biol. 2023 Oct 26;21(10):e3002341. doi: 10.1371/journal.pbio.3002341 (PMC10602308; doi:10.1371/journal.pbio.3002341)
Supplement: Attachment — Submitted filename: Bichet_Response to Reviewer Comments.docx [file pbio.3002341.s011.docx]

**Editor Comments:**

Dear Dr Barr,

Please allow me to first apologize for the delay in the processing of your manuscript. This delay is caused by my difficulty in recruiting reviewers for your manuscript. I am sorry for this unexpected event, and I thank you for your patience while your manuscript "Mammalian cells internalize bacteriophages and utilize them as a food source to enhance cellular growth and survival" was peer-reviewed at PLOS Biology. It has now been evaluated by the PLOS Biology editors, an Academic Editor with relevant expertise, and by several independent reviewers.

In light of the reviews, which you will find at the end of this email, we would like to invite you to revise the work to thoroughly address the reviewers' reports.

As you will see below, the reviewers find the manuscript interesting, but find some issues that should be solved before publication, such as overstatements regarding the adaptation. Reviewer #1 thinks that it would be good to show that your results are reproducible with other phages. We consider that given that you imply that this is generalizable, it would be helpful to add at least one other phage for comparison.

Given the extent of revision needed, we cannot make a decision about publication until we have seen the revised manuscript and your response to the reviewers' comments. Your revised manuscript is likely to be sent for further evaluation by all or a subset of the reviewers.

We expect to receive your revised manuscript within 3 months. Please email us ([plosbiology@plos.org](mailto:plosbiology@plos.org)) if you have any questions or concerns, or would like to request an extension.

At this stage, your manuscript remains formally under active consideration at our journal; please notify us by email if you do not intend to submit a revision so that we may withdraw it.

**IMPORTANT - SUBMITTING YOUR REVISION**

Your revisions should address the specific points made by each reviewer. Please submit the following files along with your revised manuscript:

1. A 'Response to Reviewers' file - this should detail your responses to the editorial requests, present a point-by-point response to all of the reviewers' comments, and indicate the changes made to the manuscript.
*NOTE: In your point-by-point response to the reviewers, please provide the full context of each review. Do not selectively quote paragraphs or sentences to reply to. The entire set of reviewer comments should be present in full and each specific point should be responded to individually, point by point.
You should also cite any additional relevant literature that has been published since the original submission and mention any additional citations in your response.

2. In addition to a clean copy of the manuscript, please also upload a 'track-changes' version of your manuscript that specifies the edits made. This should be uploaded as a "Revised Article with Changes Highlighted" file type.

Paula Jauregui, PhD,

**REVIEWS:**

**Reviewer #1**: This is a compelling Short Report manuscript, which describes a study examining internalization of phage T4 particles by cells of eukaryotic origin. The main conclusion is that phage internalization promotes growth of cells.

The paper is generally well-written and nicely organized. The results are exciting, from the standpoint of phages promoting growth as opposed to activating innate immunity of eukaryotic cells. I do not have many comments, but one of my major concerns is that the data are reported while insinuating - but not proving - that the observations indicate cellular adaptation. Here are my major and minor comments that the authors should consider:

**Major comments:**
1. The use of the phrase 'food source' seems inappropriate or unnecessary; although this may capture the attention of non-biologists or the lay public, 'food' generally refers to a substance containing protein, carbohydrate, fat etc. that nourishes a cellular metabolizing organism. But when a photosynthesizing plant uses sunlight for growth, do botanists refer to this as 'food'? The paper title could just as easily read "… internalize bacteriophages as resources to enhance cellular growth …". I suggest this change to better reflect the true biology (as I understand it), and to avoid confusion by the reader.

We thank the reviewer for their comments. We were indeed trying to capture the attention of non-biologists with this title and realize this was an overstatement. We have changed the title, along with all associated in-text statements referencing ‘food’, as per reviewer recommendation.

2. Of course, a main remaining question is WHY are the phage particles used as resources, and whether the cells are evolved via adaptation to benefit from this internalization, versus simply capitalizing on the process because the steps of internalization and downstream 'processing' of the phages are hijacked from other adaptive traits. This is not trivial to disentangle, as most purported adaptive traits are assumed rather than proven. The paper clearly hints that this is adaptive (e.g., lines 338-9: "cells are internalizing phages as a food source to maximize their growth and metabolism").

We have scaled back this claim in line with our modified title and in-text changes. We have further expanded our limitations section, noting that further research is required to disentangle this open question (see lines 386-392).

I view the data as indicating the cells capitalize on phage internalization (e.g., their nucleotides), but it seems a stretch to conclude they are tuned to use phages as food per se. I caution the authors to not overstep in their claims. Only one phage is examined, and it is unknown whether this phenomenon would work similarly for other viruses (which the authors admit).

We appreciate the reviewer’s point. We have made changes throughout the manuscript to scale back our claims, to further address the limitations and highlight the early stages of our findings.

Relatedly, why should the cells be particularly good at internalizing and using phage T4 this way, as opposed to other phages which are perpetually or occasionally present in the microbiome? On the one hand, this is a Short Report and the authors are not expected to test widely across various phages. But on the other hand, just because this is a short paper it does not justify insinuating that this is adaptive without having looked at the phenomenon more widely. Thus, I believe the authors should be very careful in how they present the findings, so that the audience does not prematurely consider this to be adaptive. Indeed, the authors admit that other non-cancerous cells should be examined (lines 425-6). Immortalized cells are convenient, but biologically 'weird'. For this reason, studies using such cells are understandably viewed with inherent skepticism and older conclusions that ignored this weirdness are being re-examined for spurious conclusions. On the authors behalf,
I would not want conclusions of the current study to be easily dismissed after publication, when viewed through a similar lens and this is my main motivation for suggesting they tread carefully.

Thank you for your thoughts and comments. Considering these points, we have made two major changes to our manuscript. The first has been addressed above and includes significant changes to the tone, pitch, and claims throughout our manuscript. Please review our track changes version of the manuscript for all these changes.

The second was to expand upon our experimental validation section to try and address the narrow data presented with only one phage across a limited number of cell lines. Due to personnel, cost, and time limitations, it was not feasible for us to perform additional microarray analyses, Western blots on cell extracts, nor to examine this phenotype within primary cell cultures. However, we were able to conduct additional experiments utilizing an improved MTT cellular proliferation assay (see methods lines 537-555) along with the inclusion of a panel of three additional phages (**Figure 3B**). Surprisingly, we found a significant increase in cell proliferation with the addition of T4 phage compared with both our filter control and a cell only control (see lines 273-286). With the additional three phages, all showed positive trends for increased cell proliferation compared with the control, however only phage NS09, a novel uncharacterized *Escherichia coli* phage, reached significance (**Figure 3B**). While these results are preliminary and do not address the prior limitations directly, we believe they expand upon the relevance and breadth of our work and provide a starting point for the field to investigate these phenomena further.

**Minor comments:**
1. Line 391. I worry that "phage T4 has most complex capsid structure of any virus" is unwarranted given our poor knowledge of the vast majority of viruses on earth. It seems better to state "of any known virus".

This statement has been removed.

**Reviewer #2:** Mammalian cells internalize bacteriophages and utilize them as a food source to enhance cellular growth and survival.

This manuscript aimed to investigate which cellular pathways of mammalian cells are triggered following non-specific ingestion of purified phage T4 using a combination of immuno-based assays and microscopy. Bichet et al., present a strong argument for mammalian cells using bacteriophages as a source of nutrients for cellular growth. They demonstrate that phage T4 are internalised via non-specific macropinocytosis, evade triggering innate immune pathways, and instead activates growth signals (i.e., AKT-dependent pathways; protein kinase B) that are associated with, for example, increases in cell metabolism and actin reorganisation. Projects such as this may yield the next generation of biotherapeutics with novel delivery systems, and I commend the authors on tackling such a study.

We thank the reviewer for their comments.

In general, I have no major comments about the results, figures, and discussion sections. The overall analyses are robust, and the conclusions are contained within the limitations of this study. However, additional minor revisions are needed to strengthen the manuscript.

**Abstract:**
Concise and well written.

**Introduction:**
The introduction is well structured and coherent. It provides sufficient background and rationale for the remainder of the manuscript. There are some points to address that I think will help further strengthen this section.

Line 89: "On the one hand, certain phages are known to induce…"
Please be specific in stating what these "certain" phages are.

We have added two examples of phages (T4 and T7) within the introduction (see lines 85-88).

Line 93: "… inflammation in response to specific phages".
Please state what these "specific" phages are.
We have added an examples of phage Pf, which is known to induce inflammatory responses (see lines 87-89).

Line 110: Please include a concluding remark (e.g., two to three sentences) on the key findings and the implications of this study.

We have included additional sentences at the end of the introduction (see lines 102-105)

**Results:**
The results section reads more like a narrative/thesis and some text can be either moved to the discussion section or removed entirely.

We have made significant changes both to this section, and throughout the entire manuscript, to both remove text and improve the clarity and readability of our writing. Please see the attached track changes version of the manuscript for all these changes.

For example, Lines 141 - 149 can be moved to the discussion section: "Once internalized, phages and their nucleic acids could be recognized by the nucleic acid receptor TLR9, a transmembrane protein that resides within endocytic vesicles and preferentially binds DNA from bacteria and viruses. TLR9 is expressed mainly in immune cells (including leukocytes and macrophages) but is also known to be expressed in a range of other cell types, including A549 cells (36). For TRL9 to be activated within the macropinosomes, phage DNA would first have to escape the phage capsid. This could happen through the triggering of the phage ejection apparatus or phage degradation due to acidic conditions found within the endosome (22). Conversely, if the phage particles stay intact, then the phage DNA would not be accessible and TLR9 should not be activated."

We have significantly rewritten this section along with the entirety of the results, moving some of the above content to the discussion, and other parts to the methods to improve readability.

Line 140: Describe instead what is observed in Figure 1.

This section has been rewritten to explain our observations (see lines 133-136)

Can the authors quantitate the concentration of phage that have been internalised across the three timepoints using the images already collected? E.g., Jonkman et al., Tutorial: guidance for quantitative confocal microscopy. Nature Protocols.  <https://doi.org/10.1038/s41596-020-0313-9>

This is not entirely feasible as a single fluorescence-labelled phage is not visible within the background fluorescence of the mammalian cell using the microscopy facilities we have access to. Instead, the fluorescent puncta that we are visualizing are composed of large aggregates of many fluorescence-labelled phages. So, while it is feasible to provide a qualitative overview of fluorescence-associated signal using the above protocol, quantitative measurements using this method are flawed within our study system.

Our previous publications (Bichet at al., 2021, iScience), which are cited in text, have quantified phage internalization using these approaches, including qualitative fluorescence values matched with quantitative molecular measures.

Finally, the fluorescence images were included in this manuscript more to serve as visual confirmation that mammalian cells are internalizing T4 phage particles, rather than as a quantitative measure.

Lines 131 - 134: Please include some basic stats of the extracted DNA. Extraction kits will fragment and may alter the form of DNA that is different to in situ. I think information on the "Phage DNA" sample would be useful (as supplementary data) for readers to know quality of DNA and size of DNA fragments.

We have added a short description in text on the DNA extraction method and fragment size (see lines 127-129). Further details are found within the methods.

Line 131: Similar to above, can the authors provide information on phage proteins in the "Capsid-only" sample? Are there structural details that are lost through in vitro processing, which would otherwise trigger immune responses?

We have added further details on our capsid-only sample in text (see lines 123-126), although we have not performed a detailed structural analysis on this sample.

Lines 215 - 221: Please move to methods section. What other pathways were activated?

This paragraph was moved to the Microarray method (lines 491-496).

Many other pathways were activated due to the presence of phages (please see Supplemental Figure 2 & 3). We chose to focus on pathways that were prominent in our network analysis and shared between samples to keep the scope of the paper clear and concise. Our microarray data is available in our supplementary materials for further analysis.

Lines 222 - 225: "From this analysis, we found 52 hits for the MDCK-1 cells and 150 hits for the A549 cells, which utilized the improved KAM-2000 antibody array. Based on this analysis, we focused our attention on two main pathways - AKT and CDK1 - the were common across the two antibody microarrays." I would like to see a summary plot - either a Venn diagram or upset plot - highlighting the number of hits that were unique and common between the two mircoarrays; also, a more clear statement for rationale in focusing on AKT and CDK1 pathways.

We have made several plots and Venn diagrams to visualize these overlaps as per the reviewer suggestions (see Figures below). However, there are several points to consider with regards to this approach that we would like to highlight. First, during our experiments, Kinexus, who is the supplier of the microarray, updated the KAM-1325 array to the improved KAM-2000 array (see lines 175-181 for description). This complicates the requested Venn/Upset analyses as not only is the KAM-2000 array expanded, but some of the original antibodies were changed making direct comparison between the datasets difficult. Second, while we did see the activation of CDK1 and AKT pathways between the A549 and MDCK-I cell lines through our pathway analysis (see lines 183-188 for description), these were often activated via different phosphorylation sites and hence, were detected by different antibodies within the microarray. As a result, when we performed the reviewer’s request of a direct mapping comparison between antibody hits from our datasets (see lines 188-191), which include 52 hits to MDCK-1 cells and 150 hits for A549 cells, we found only four antibody hits that were shared between our two datasets. However, this direct comparison is not an accurate representation of the interaction network due to the issues we describe above.

To address this, we compared the unique and common interactions between the two datasets based upon the phosphorylation network, in which kinases and substrates are interacting. This is a higher-level analysis than the individual antibody comparison described above. From this interaction analysis, we find 37 interactions from the MDCK-1 cells and 110 interactions from the A549 cells. Breaking this down into kinases and substrates, we find 7 shared kinases between the two datasets (**Figure R1 & R2**) and 6 shared substrates (**Figure R3 & R4**). Importantly, from this interaction re-analysis, we show that both the CDK1 and AKT pathways were common for both kinase and substrate interactions across both datasets, providing further evidence on the relevance of our pathway analysis approach (see lines 183-188 for description). We do note that there were other shared interactions in both kinases and substrates that were not directly discussed/analyzed in our manuscript. Our analysis is not meant to be exhaustive and there are likely additional cellular responses that could be further analyzed and investigate in later studies.

**Figure R1.** Venn diagram of kinase interactions between A549 and MDCK-1 datasets.

**Figure R2.** Barplot showing both unique and common interactions from kinases found in A549 and MDCK-1 datasets.

**Figure R3.** Venn diagram of substrate interactions between A549 and MDCK-1 datasets.

**Figure R4.** Barplot showing both unique and common interactions from substrates found in A549 and MDCK-1 datasets.

We would further like to emphasize the difference in our pathway analysis approach compared with the traditional fold-change analyses of single antibodies. Our approach was designed to identify long interaction networks of phosphorylation events and kinase-substrate interactions, with the rationale being that longer pathway interaction networks provide greater biological relevance than single antibody fold-changes. Through this analysis, we identified the AKT and CDK1 pathways were associated with deep network pathways, had multiple activators and downstream effectors, and had shared activation profiles across both datasets. It was for these reasons that we chose to focus on these two pathways. We have added an expanded rationale for this focus (see lines 190-194).

Supplementary Figures 2 and 3 depicting the network analyses is a crucial piece of data that I think should be included in the main text.

As suggested by the reviewer, the two supplementary figures S2 and S3 have now been added to the main text in **Figure 2** panels A and B.

Lines 251 - 255: Please move to discussion section. "Macropinocytosis, which is the process T4 phage utilize to access the cell, requires significant actin reorganisation to generate the membrane ruffles and cell-to-cell membrane fusion to form macropinosomes. As such, the activation of Ezrin through AKY may lead to a positive feedback loop resulting in enhanced phage uptake through the micropinocytosis pathway."

This paragraph has been reworked and moved to the discussion section (lines 343-348).

Lines 270 - 271: Please rephrase to read "With the reduction in activation of JUN, the progression through the G1 phase of the cell cycle would be delayed…"

The text has been modified accordingly (lines 237-239).

Lines 281 - 286: Please move to discussion section.

This section has been reworked and significantly shortened (lines 246-248).

Lines 293 - 301: Consider moving to discussion section.

This section has been reworked and moved to the discussion section (lines 342-348).

**Discussion:**
Lines 425 - 426: What are some of the other limitations to this study? In vitro vs., in situ. How can the results obtained in vitro be extrapolated for use with human samples?

More precision on the limitation and future implications of our study have been added to the concluding paragraph (lines 380-392).

Line 433: Please include a concluding statement emphasising the importance of current findings. This is study represents an important step forward in better understanding the impacts of phage biology in human health - with implications in cancer biology, immunology, and the microbiome sciences.

A concluding sentence has been added at the end of the discussion (lines 390-392)

**Methods:**
The choice of cell lines is not made clear. What was the rationale behind the choice of cell lines used in this study?

The reason for the choice of these cell lines for this study has been specified in the method section: “The choice of A549 and MDCK-I cells has been made following the results obtained in a previous study (14), showing the internalization of T4 phages in both these cell lines.” (lines 424-425).

Lines 454 - 455: Missing comma. Please rephrase to read: "We prepared a new comparative control sample using ultra-pure T4 phage lysates."

The text has been rewritten (lines 413-414).

Alternatively, the copy editor at the journal should go through and correct the minor grammatical and syntax errors.

We have made significant efforts to rewrite the manuscript and improve these grammatical and syntax errors.

Lines 510 - 512: "The day before the phage treatment…" and "The day after 2 ul of…"
Please clarify if "the day before/after" refers to a 12 h or 24 h period, or otherwise

We specified the time frame as suggested by the reviewer (lines 475-479).

Lines 518 - 532: The approach taken for the two Ab microarrays are very similar. Please merge as one subsection.

The two microarray methods were merged as one (lines 483-496).

Line 543: "…was considered as an activation effect for this analysis All nodes (kinases) without a directed..."
I think there is a period missing between "analysis" and "All"

The missing period has been added to the text (line 507).

Line 562: "The next day, phages at…".
Please clarify the number of hours.

The time frame has been specified in the text (line 520).

Line 566: "Cells were quickly centrifuged before…".
Please provide the speed and time of centrifugation step.

The centrifugation details have been added to the method (line 526-527).

Line 570: What does PI stand for.
It stands for Propidium Iodide. We added the description of PI in the text (line 528).

**Figures:**
Figure 2: Please clarify what the error bars represent.

The description of the error bars has been added to the figure legend – note this is now Figure 3.

Figure 3A and 3B: Under the "End Result" subheadings, replace red crosses, downward red arrows, and upward green arrows with bullet-pointed text e.g., "No TLR9 activation", "Decreased apoptotic activity", and "Increased cell metabolism & cell survival".
The red and green arrows might confuse readers into looking for them in graphic.

The figure has been modified accordingly – note this is now Figure 4.
